# Gelation of Hole Transport Layer to Improve the Stability of Perovskite Solar Cells

**DOI:** 10.1007/s40820-023-01145-y

**Published:** 2023-07-10

**Authors:** Ying Zhang, Chenxiao Zhou, Lizhi Lin, Fengtao Pei, Mengqi Xiao, Xiaoyan Yang, Guizhou Yuan, Cheng Zhu, Yu Chen, Qi Chen

**Affiliations:** Beijing Key Laboratory of Construction Tailorable Advanced Functional Materials and Green Applications, MIIT Key Laboratory for Low-Dimensional Quantum Structure and Devices, Experimental Center of Advanced Materials, School of Materials Science and Engineering, Beijing Institute of Technology, Beijing, 100081 People’s Republic of China

**Keywords:** Perovskite solar cell, Hole transport layer, Gelation, Humidity stability, Aggregation of LiTFSI

## Abstract

To achieve high power conversion efficiency (PCE) and long-term stability of perovskite solar cells (PSCs), a hole transport layer (HTL) with persistently high conductivity, good moisture/oxygen barrier ability, and adequate passivation capability is important. To achieve enough conductivity and effective hole extraction, spiro-OMeTAD, one of the most frequently used HTL in optoelectronic devices, often needs chemical doping with a lithium compound (LiTFSI). However, the lithium salt dopant induces crystallization and has a negative impact on the performance and lifetime of the device due to its hygroscopic nature. Here, we provide an easy method for creating a gel by mixing a natural small molecule additive (thioctic acid, TA) with spiro-OMeTAD. We discover that gelation effectively improves the compactness of resultant HTL and prevents moisture and oxygen infiltration. Moreover, the gelation of HTL improves not only the conductivity of spiro-OMeTAD, but also the operational robustness of the devices in the atmospheric environment. In addition, TA passivates the perovskite defects and facilitates the charge transfer from the perovskite layer to HTL. As a consequence, the optimized PSCs based on the gelated HTL exhibit an improved PCE (22.52%) with excellent device stability.

**Supplementary Information:**

The online version contains supplementary material available at 10.1007/s40820-023-01145-y.

## Highlights

- The gelation of hole transport layer generates a dense and uniform hole transport layer film and significantly inhibits the aggregation of lithium bis(trifluoromethane sulfonyl)imide in spiro-OMeTAD.
- The gelated hole transport layer confers enhanced charge carrier transport and better humidity and operational stability of perovskite solar cells.

**Supplementary Information:**

The online version contains supplementary material available at 10.1007/s40820-023-01145-y.

## Graphical abstract

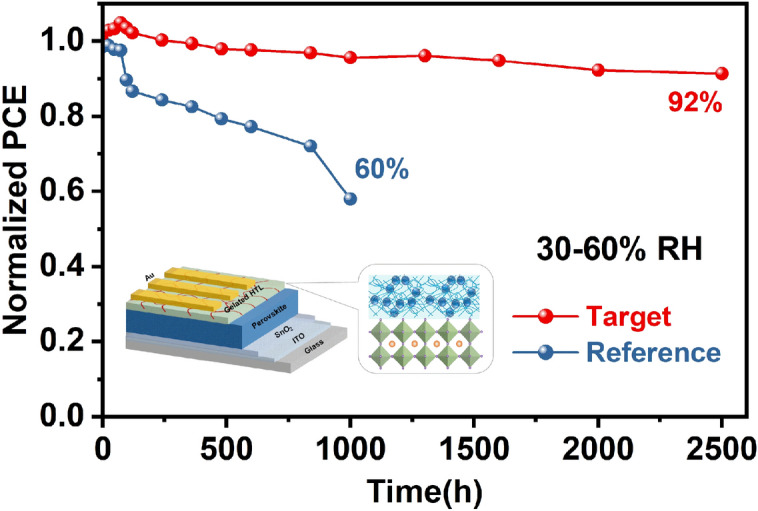

## Introduction

Organic–inorganic perovskite solar cells have made rapid progress in recent years due to their incredible optoelectronic properties, such as high optical absorption coefficient, long carrier diffusion length, and low exciton binding energy [1–7]. In recent ten years, the certified power conversion efficiency (PCE) of perovskite solar cells (PSCs) increased from 3.8% [2] to more than 25% [8] which can rival crystalline silicon cells. But until recently, issues with long-term instability in the actual working environment have prevented PSCs from commercializing [9]. Except for the vulnerability of perovskite layers toward ambient conditions, the most frequently used small-molecule hole-transporting layer (HTL) for highly efficient PSCs, spiro-OMeTAD (2,2′,7,7′-tetrakis(N,N-di(4-methoxyphenyl)amino)-9,9-spirobifluorene), shows poor stability toward humid conditions [10].

The virgin spiro-OMeTAD is poorly electrically conductive; hence, dopants are typically employed to increase hole mobility [11–13]. One typical addition to increase conductivity is lithium bis(trifluoromethane sulfonyl)imide (LiTFSI) [14]. Unfortunately, in humid environments, the hydrophilic lithium salt would hasten the device’s deterioration [15, 16]. Additionally, the devices’ *J-V* hysteresis effect could be brought on by Li^+^ migration [17]. The PSCs are destroyed when LiTFSI aggregates and hydrates as a result of exposure to the ambient environment [18–20].

Thus far, many efforts have been devoted to optimizing the HTL of PSCs for enhanced PCE and stability. Major efforts have been exerted to explore new HTL materials [21–23]. To counteract the infamous effect of Li ions, novel dopants such as spiro-OMeTAD^2+^(TFSI^–^)_2_ [24, 25], lithium-ion endohedral fullerene [26], and lithium-ion-free salts [27–29] have been investigated. Alternative doping approaches [30, 31] and other effective p-dopants for HTL [32, 33] also have been developed to address these shortcomings. These attempts successfully enhance device stability, but the PCE of the corresponding device is generally not better than that of conventional lithium salts. For both spiro-OMeTAD and other prospective HTLs, there is an urgent need to decrease the harmful effects of dopants while maintaining device performance.

When reviewing the previous work, we find these contributions mainly focus on the replacement of only one single component, ether matrix or additive, to make the mixture compatible. Alternatively, it would be feasible to immobilize the additives in the matrix by tailoring their interaction. Herein, we propose a strategy to dope thioctic acid (TA), along with the LiTFSI and tBP as additives, into spiro-OMeTAD to form a gel. Our work finds that TA interacts with lithium salt to form the gelated HTL, which inhibits the agglomeration of LiTFSI and consequent voids in the film. The compact film also improves the stability of beneath perovskite film against moisture invasion. Additionally, it can also passivate the surface defects of perovskite, which confers enhanced charge carrier transport and interfacial stability at perovskite/HTL. As a result, we achieved a champion PCE of 22.52% in the PSCs which also exhibited much improved stability particularly under humidity conditions. The PSCs maintained 92% of its initial PCE after aging for 2500 h at room temperature in ambient air conditions.

## Materials and Methods

### Materials

In this article, materials used in experiments as received without further purification, including cesium iodide (CsI, 99.9%, Sigma-Aldrich), lead iodide (PbI_2_, Xi’an Polymer Light Technology), methylammonium chloride (MACl, Xi’an Polymer Light Technology), materials for charge transporting layers (SnO_2_ (15 wt% colloidal dispersion, Alfa), 2,2’,7,7’-tetrakis-(N,N-di-4-methoxyphenylamino)-9,9’-spirobifluorene (spiro-OMeTAD, Xi’an Polymer Light Technology), bis(trifluoromethane)sulfonimide lithium salt (LiTFSI, 99.95%, Sigma-Aldrich), thioctic acid (TA, 99%, Sigma-Aldrich). The solvents, including Chlorobenzene (CB, Sigma-Aldrich, 99.9%), N,N-dimethylformamide (DMF, 99.99%, Sigma-Aldrich), dimethylsulfoxide (DMSO, 99.5%, Sigma-Aldrich), isopropanol (99.99%, Sigma-Aldrich), acetonitrile (ACN, 99.95%, Sigma-Aldrich), tBP (99.9%, Sigma-Aldrich)). Besides, formamidinium iodide (FAI, Dyesol) was further purified after purchasing.

### Device Fabrication

The ITO substrate was cleaned with ultrapure water, acetone and ethanol in an ultrasonic system each for 30 min. Before deposition, the ITO substrate was dried with high purity N_2_ gas and treated with ultraviolet–O_3_ for 30 min to improve its wettability. A compact SnO_2_ layer was deposited by spin coating a 1:6 diluted SnO_2_ colloid aqueous solution at 4000 rpm for 30 s, followed by annealing at 150 °C for 30 min in air. Before transferring into a glove box for perovskite film deposition, the substrates were treated with UV light for 10 min. The PbI_2_ precursor was prepared by dissolving 691.5 mg PbI_2_ and 13 mg CsI in 1 mL mixture solvent of DMF: DMSO (V: V = 9:1) and stirred at 70 °C for 5 h. For organic cation precursors, 90 mg of FAI and 9 mg of MACl were dissolved in 1 mL of isopropanol and then continuously stirred for 30 min at room temperature. All mixed-solutions were filtered with a 0.22 μm polytetrafluoroethylene (PTFE) filter. The perovskite films were prepared by a typical two-step solution process: The PbI_2_ precursor was spin-coated on the SnO_2_ substrate in a N_2_ glove box at 2300 rpm for 30 s (accelerated speed 3000 rpm s^–1^) and then annealed at 70 °C for 1 min. After cooling to room temperature, the organic cation precursor was spin-coated on the PbI_2_ film at 2900 rpm for 30 s (accelerated speed 3000 rpm s^–1^) in a N_2_ glove box. Then, the films should be transferred from the glove box to the hot plate in the atmosphere (20–30% relative humidity) as soon as possible for annealing at 150 °C for 10 min, and the mild moisture is required to obtain high-quality perovskite films. After cooling down, a hole transport layer was spin coated on the perovskite film at 3000 rpm for 30 s. Two kinds of HTL precursors were used. For the Reference HTL deposition, a precursor solution consisted of 72.3 mg spiro-OMeTAD, 20 μL TBP and 35 μL of LiTFSI (260 mg mL^–1^ in acetonitrile) in 1 mL CB. For the Target HTL deposition, the components in the precursor solution include 72.3 mg spiro-OMeTAD, 1 mg TA, 20 μL TBP and 35 μL of LiTFSI (260 mg mL^–1^ in acetonitrile) in 1 mL CB. After oxidation overnight, a 100-nm-thick Au film was deposited by thermal evaporation as the back contact. The device area was defined and characterized as 0.0805 cm^2^ by metal shadow mask.

### Characterization

The rheological properties of poly(TA) were determined by measuring their storage modulus (G′) and loss modulus (G″) using an Anton Paar instrument (Physica MCR 301, Germany) equipped with a parallel-plate geometry (15 mm diameter). The strain sweep measurements of G′ and G″ as a function of angular strain at frequency = 0.5 Hz were carried out at 25 °C in the sweeping strain range from 0.1 to 2500%. The temperature sweep measurements of G′ and G″ were carried out form 25 to 100 °C at strain of 1% and frequency of 0.5 Hz. The Fourier-transform infrared spectroscopy (FTIR) was obtained by Magna-IR 750 (Nicolet, USA). ^1^H NMR spectra were measured using a Bruker AVANCE III 300 MHz NMR Spectrometer in the designated deuterated solvent. The XPS data were obtained by Axis Ultra XPS spectrometer (Kratos, U.K.) with Kα radiation of Al, and operated at 225 W. The scanning electron microscopy (SEM) was acquired by Hitachi Regulus 8230. Nano-FTIR experiments were carried out at the Bruker Dimension Icon IR using the PRUM-TNIR-D-10 prob. The resonant frequency of the prob is 320 kHz, and the elastic constant is 42 Nm^−1^. ToF–SIMS measurement was performed using a PHI NanoTOF II instrument (ULVAC-PHI, Inc.), a 30 keV Bi^+^ pulsed primary ion beam was used for the analysis. The UV–vis absorption spectra of the samples were obtained by a UV–visible diffuse reflectance spectrophotometer (UV–vis DRS, Japan Hitachi UH4150). The 2D PL mapping was executed by a laser scanning confocal microscope (Enlitech, SPCM-1000) equipped with 470 pulse laser and galvo-based scanner. X-ray diffraction (XRD) data were measured using a Bruker D8 Advanced, equipped with Cu K*α* radiation (*λ* = 1.5406 Å). The steady-state PL and TRPL were obtained by FLS1000 (Edinburgh Instruments Ltd) equipped with a 450 W Xe lamp using the excitation wavelength of 470 nm. The photovoltaic performance of the PSCs was collected with a source meter (Keithley 2400) at glovebox under AM1.5G illumination at 1000 W m^−2^ solar simulator (SS-F5-3A, Enlitech). The *J-V* scan method was by reverse scanning from 1.2 to − 0.2 V or forward scanning from − 0.2 to 1.2 V at a scanning speed of 50 mV s^−1^. No precondition before testing for all devices. The shading mask was sent to the National Institute of Metrology, China, for certification and the active area was defined as 0.0805 cm^2^. All the *J-V* measurements were conducted under Xeon lamps, and only MPP tracking experiment and the photo-stability experiment used LED white light lamps for aging. The external quantum efficiency (EQE) curve was recorded by an Enli Technology (Taiwan) EQE measurement system. A calibrated silicon diode with the known spectral response was used as a reference.

## Results and Discussion

### Gelation of Hole Transport Layer

**Fig. 1:**
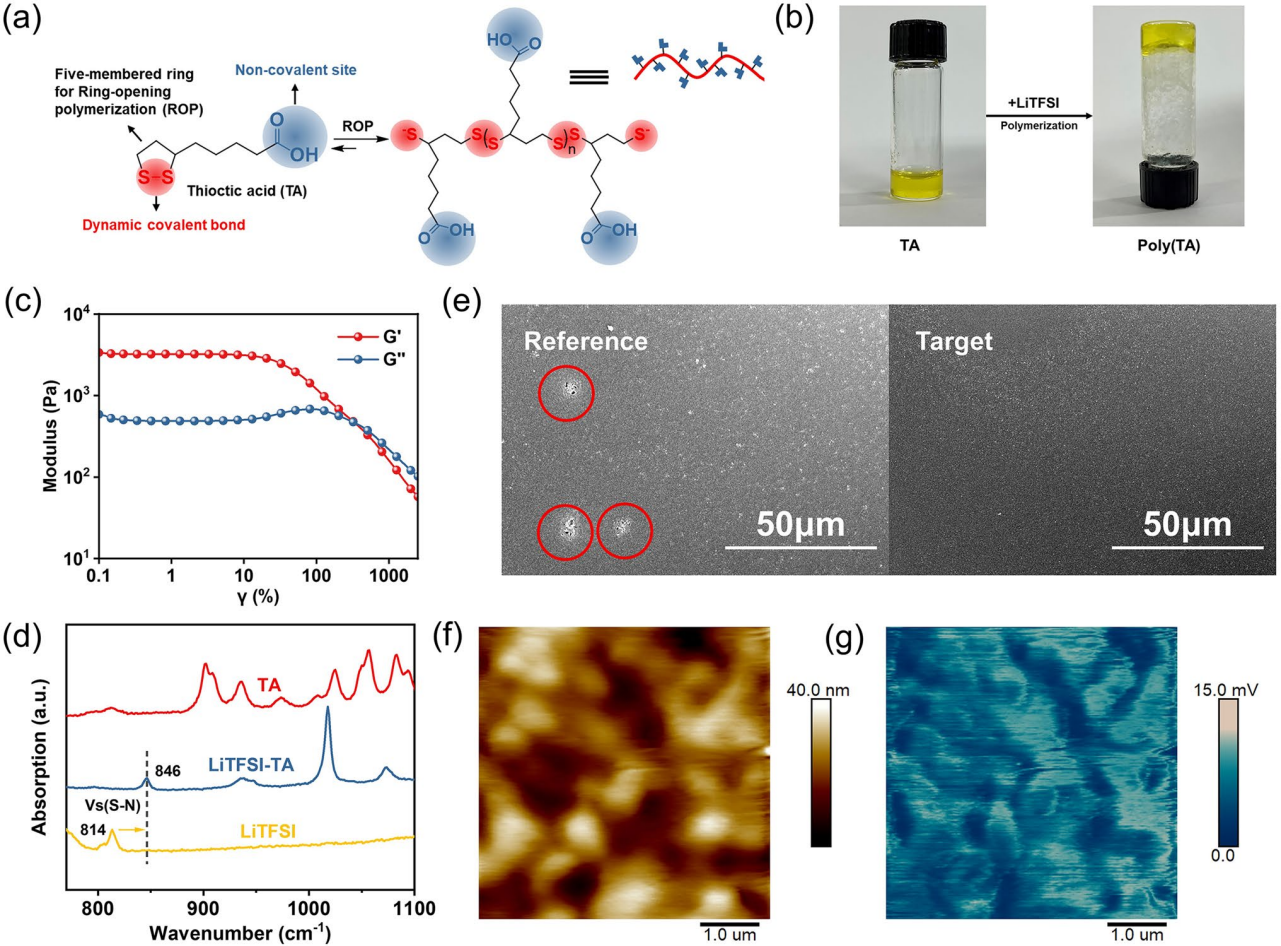
**a** Schematic representation of crosslinking polymerization of TA. **b** The pictures of the polymerization of the TA. **c** Storage modulus (G′) and loss modulus (G′′) for poly(TA) gels on strain sweep. **d** FTIR spectra of TA (red), mixture of LiTFSI and TA (blue), LiTFSI (yellow). **e** Scanning electron microscopy (SEM) images of spiro-OMeTAD and spiro-OMeTAD doped with TA films. **f** AFM images of Target film and **g** corresponding Nano-FTIR images. Nano-FTIR at an IR frequency of 1693 cm^–1^ (which is resonant with the C = O stretching absorption of TA)

TA is a naturally existing small molecule and acts as an essential coenzyme for aerobic metabolism in animals, which contains hydrophobic 1,2-dithiolanes and alkyl chain groups as well as hydrophilic carboxylic acid groups [34]. This molecule contains two forms of dynamic chemical bonds: dynamic covalent disulfide bonds and noncovalent hydrogen bonds (H-bonds) of the carboxyl group. This unique molecular structure makes TA a potential candidate for the construction of robust continuous network as a crosslinker (Fig. 1a) [35]. TA is dissolved in chlorobenzene to form a yellow clear solution. When LiTFSI is added to TA solution, a yellow transparent gel-like polymer network formation occurs in just a few minutes (Fig. 1b). This transformation is attributed to the formation of a cross-linked polymer known as poly(TA) [36].

The gelation behavior was revealed using rheological measurements. According to Fig. 1c, a strain sweep test was performed over a wide strain range (*γ* = 0–2500% strain). At about 340% oscillatory strain amplitude, it was found that the dramatic drop in G′ occurred more quickly than that in G′′, indicating the occurrence of the gel to sol transition, in which the gel tends to change into the liquid phase. Since both stable storage and loss modules were detected prior to the critical strain (*γ* = 50%), the gel network was unaffected, the gel failure occurs at the crossover point of G′ and G′′ approximately *γ* = 340%. Though being cross-linked, a reversible solid-to-liquid transition at 50 °C could be observed by rheological analysis in Fig. S1, in which G″ surpassed G′ above 50 °C. This supramolecular polymer is dynamically reversible, as evidenced by the gel’s lability to concentration drop and temperature increase, which would cause the substance to turn into a viscous polymer solution following heating or dilution by water [37]. Disulfide backbones and H-bonding cross-linkers both have dynamic properties that may be responsible for this phase transition. Disulfide linkages may be broken by high temperatures, which then depolymerized the zwitterionic elastomer [38]. This transition temperature could be improved by increasing the concentration of monomer solution concentration to favor intermolecular reactions and adding metal ions, such as Fe^3+^, Pb^2+^, Zn^2+^, and Ca^2+^ to stabilized poly(TA) network [35]. Moreover, the mechanical property of the gel was analyzed by tensile test (Fig. S2). Typical tensile curves of the gel are presented in Fig. S2b. It further confirmed the formation of gel in this system.

FTIR was performed to investigate the coordination between the TA and LiTFSI, which construct the crosslink structure. As shown in Fig. 1d, S–N in pure LiTFSI shows a typical symmetric stretching vibration mode at 814 cm^−1^ [39] which shift to 846 cm^−1^, when mixed with TA. The downward shift of the S–N symmetric stretching vibration frequency of 32 cm^−1^ demonstrates a strong interaction between TA and LiTFSI. Based on the natural amphiphilic structure of the TA molecule, the carboxyl groups of TA are easily deprotonated. Then, the chemical reaction between TA and Li^+^ results in the formation of Li-TA thiolate. TA is chemically polymerized via ring opening due to the presence of Li-TA thiolate which acts as an initiator for the ring opening polymerization of TA (Fig. S3) [40]. Figure S4 shows the full FTIR spectra of TA, LiTFSI and the TA-LiTFSI mixture. ^1^H-NMR spectroscopy of the mixture of TA and LiTFSI is shown in Fig. S5. It can be seen that the mixture of TA and LiTFSI compared with the original TA molecule, the active hydrogen on TA has significantly shifted. This indicates that the active hydrogen in these locations is attracted by the TFSI^−^. X-ray photoelectron spectroscopy of the pure LiTFSI film and TA-LiTFSI mixture film was conducted (Fig. S6). The remarkable shift in the Li 1 s peak of the film to lower binding energy after doping TA into LiTFSI indicates that a strong interaction occurred between LiTFSI and the TA molecules.

Further, TA was added into the composition of HTL to make the gel. As shown in Fig. S7, the HTL precursor solution immediately became gel while adding TA into solution. Generally, the HTL based on spiro-OMeTAD is deposited on the perovskite by spin coating. Obviously, the gel state cannot be directly applied by spin coating. In order to prepare HTL layer, suitable solvent was selected to dissolve the gelation precursor. It was found that volatile formic acid or ethanol can dissolve the gel. When we added a small amount of solvent, the gel can be completely dissolved. After dissolution of gelation precursor, HTL could be prepared on perovskite films by spin coating. Spiro-OMeTAD (*M*_*w*_ = 1225) is a kind of small molecule. In contrast, poly(TA) is a kind of cross-linked polymer and is easy to form uniform and condense film. As such, the gelated HTL can favor the spiro-OMeTAD film formation process. To further investigate the role of TA in governing the morphology and microstructure of the gelated HTL film, SEM and optical microscopy are conducted. The spiro-OMeTAD HTL without TA hereafter denoted “Reference” and the HTL doped with TA hereafter denoted “Target”. Reference film presents some bright spots (highlighted by red circles) in the SEM image (Fig. 1e). We speculate that the bright spots are attributed to the aggregation of LiTFSI [41]. In contrast, such bright spots disappear in the Target, which shows uniform and dense surface (Fig. 1e). Moreover, we confirm that the introduction of TA significantly improves the film morphology according to the AFM results (Figs. 1f and S8a). The Reference film shows round-shaped aggregates in the film with the root-mean-square (R_MS_) roughness of 8.05 nm (Fig. S8a). In the Target sample, however, the film exhibits smooth and flat surface with R_MS_ decreased to 5.73 nm (Fig. 1f). The spatial distribution of TA was verified by atomic force microscopy-based infrared spectroscopy (AFM-IR) by mapping the characteristic IR vibrational signal of C = O at 1693 cm^−1^ (Fig. 1h). The characteristic IR signals are evenly distributed of the film, while there was no signal in Reference films (Fig. S8b). We can conclude that the gelation of HTL can prepare more uniform and condense films.

### Improved Humidity Stability

**Fig. 2:**
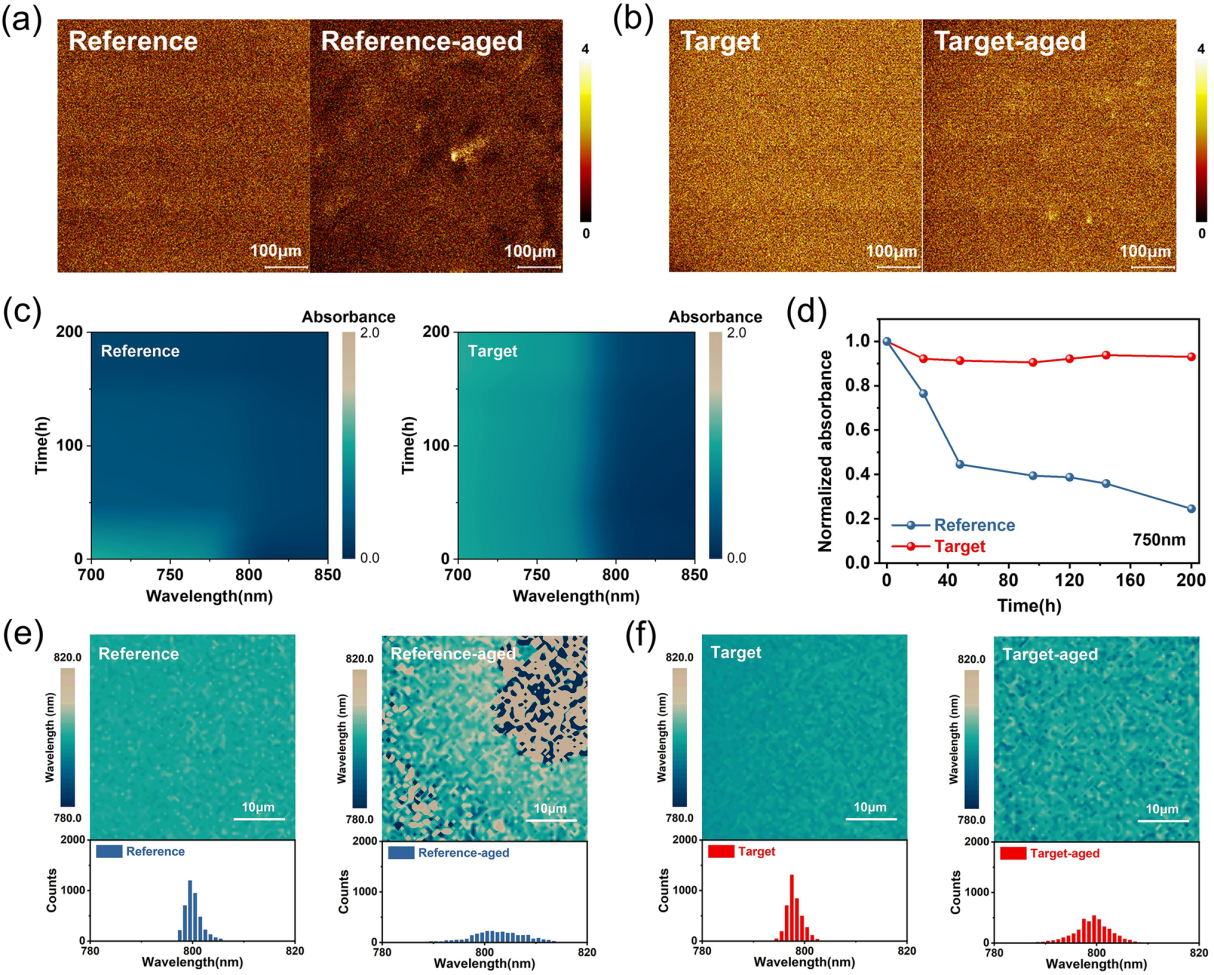
**a** 2D ToF–SIMS elemental mapping of Li^+^ of the **a** Reference film and **b** Target film before and after aging under high RH of 85–90% at 25 °C for 200 h. **c** UV–vis absorption spectra for Reference and Target perovskite films at 700–850 nm over time. **d** The normalized absorption at 750 nm of Reference film and Target film. **e** PL peak position mapping and statistical diagram of Reference. **f** Target films before and after aging under high RH of 85–90% at 25 °C for 500 h

To observe the distribution of LiTFSI in the gelated HTL film by TA addition, time-of-flight secondary ion mass spectrometry (ToF–SIMS) mapping was used to assess compositional distribution in the plane directions of the HTL (Fig. 2a, b). It is observed that the distribution of LiTFSI is uniform of both the fresh Reference film and Target film initially. However, after exposure under high relative humidity (RH) of 85–90% at 25 °C for 200 h, obvious LiTFSI aggregation occurred on the surface of the Reference film, while mitigated LiTFSI aggregation was observed in the Target film. It indicates that the gelated HTL is more robust under high humidity conditions. Moreover, the interactions between TA and LiTFSI would retard the Li aggregation as shown in the images of AFM-IR (Fig. S9) and the results of depth profile ToF–SIMS result in Fig. S10. We conducted AFM-IR measurement for the spiro-OMeTAD/tBP/LiTFSI film with and without adding TA after being aged under high RH of 85–90% at 25 °C for 200 h. The AFM-IR absorption images were collected at 1110 cm^−1^ which corresponded to the C–F stretching vibration in LiTFSI [42] (as shown in Fig. S9). It is observed the gelated HTL film exhibits a much more uniform distribution of LiTFSI, indicating the gelation of HTL could effectively retard the LiTFSI aggregation. Depth profile ToF–SIMS measurement was carried out to further study the effect of gel on the migration of Li in the device. Both Reference and Target devices with a structure of ITO/SnO_2_/perovskite/spiro-OMeTAD(TA)/Au were aged under high RH for 200 h. As depicted in the depth profile illustrated in Fig. S10, there is less amount of Li in the perovskite/SnO_2_ interface, and more uniform distribution Li in the gelated HTL layer for the Target device compared with that of the Reference, indicating that gelation of HTL could effectively retard the Li migration, which is consistent with the AFM-IR result.

Moreover, we investigate the effect of the gelated HTL strategy on humidity stability of perovskite film. Perovskite films coated with HTL were aged in humid air (RH of 85–90% at 25 °C for 200 h), and the UV–vis absorption was monitored to check the degree of film degradation. As shown in Fig. S11, both films showed a similar high absorbance in the beginning (Fig. S11a). However, after 200 h of exposure to humid air, the absorbance of the Reference film dropped sharply (Fig. S11b), while the Target film showed negligible change (Fig. S11c). Figure 2c illustrates the UV–vis absorption spectra for those films at 700–850 nm over time. For Target film, almost no decrease in the absorbance was observed after 200 h in humid air. In contrast, the Reference film exhibited a drastic decrease in light absorbance 700–850 nm after 24 h. Figure 2d shows the normalized absorption at 750 nm of Reference film and Target film. We also conducted XRD measurements (Fig. S12). The Reference film degraded into PbI_2_ (e.g., at 12.8°) and photoinactive δ-phase (e.g., at 11.7°) after 200 h in humid air. In contrast, the Target film illustrated a retarded α-to-δ phase transition with a lower peak that appeared at 11.7°.

The degradation of perovskite films after aging under high RH of 85–90% at 25 °C for 500 h was further probed by photoluminescence (PL) mapping. Both the Reference and Target films showed homogenous PL peak positions at first. However, the Target films displayed a narrower PL wavelength range after aging, in contrast to the Reference that displayed a larger wavelength range with greater heterogeneity (Fig. 2e, f). It suggested that the gelated HTL can retard the decomposition and phase transition of perovskite film by preventing water invasion. Moreover, in order to study the universality of this strategy, we also apply the gelated HTL strategy to PTAA. The results obtained are similar to those obtained by spiro-OMeTAD. After gelation of HTL, the humidity stability of Target films was exactly improved (Fig. S13). Based on the above results, we conclude that the humidity stability of perovskite films covered by the gelated HTL is significantly improved. Contact-angle measurement was carried out to determine the hydrophobicity of the HTL film. The water droplet contact angle on the gelated HTL film (67.7°) is larger than that on the reference film (52.3°) (Fig. S14), indicating reduced hygroscopicity. The decrease in hygroscopicity may be one of the reasons for the improvement of film humidity stability.

### Improved Performance and Stability of Device

**Fig. 3:**
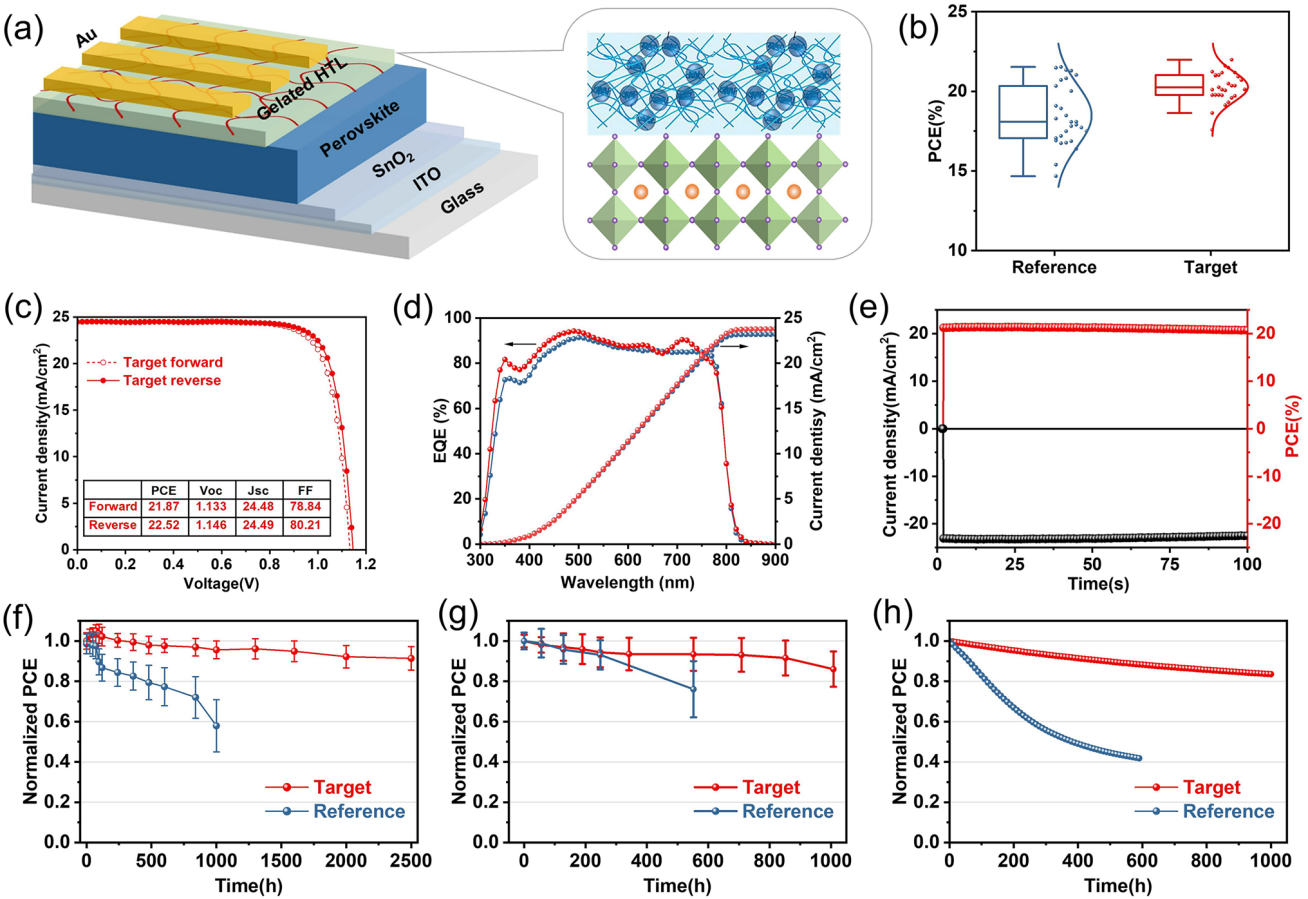
**a** Structure of PSC and the interface between perovskite and gelated HTL. **b** The statistical distributions of PCE of the Reference and Target devices. **c** *J-V* curves for the best performing target device with aperture areas of 0.0805 cm^2^. **d** EQE curve and its integrated *J*_SC_ curve of Reference and Target device. **e** The corresponding stabilized power output data at bias voltages (1.00 V) near the maximum power point. Normalized PCE evolution of the Reference and Target devices under **f** ≈30–50% RH, **g** 85–90% RH and **h** continuous illumination at MPP condition

Next, the effect of the gelated HTL on the photoelectric performance and stability of the device is investigated. To examine the photovoltaic performance of devices based on the gelated HTL, we fabricated n-i-p type planar solar cells with the architecture of ITO/SnO_2_/perovskite/spiro-OMeTAD(TA)/Au. Figure 3a shows the structure of PSC and the interface between perovskite and the gelated HTL. Figure S15 shows cross-sectional SEM images of the device. The PCE histogram of Reference and Target devices is plotted in Fig. 3b. The Target devices exhibited a higher average PCE, which increased from 18.11 to 20.22%. They also displayed a narrower PCE distribution, meaning a good reproducibility, perhaps because of the improved microscopic homogeneities and compactness of the gelated HTL film. As shown in Fig. 3c, the best Target device delivered a PCE of 22.52%, with a *V*_OC_ of 1.146 V, a *J*_SC_ of 24.49 mA cm^−2^, and an FF of 80.21%, which are all higher than the Reference devices (Fig. S16). As shown in Fig. 3d, the corresponding integrated *J*_SC_ value obtained from the external quantum efficiency (EQE) spectra was 23.78 mA cm^−2^, which was similar with the *J*_SC_ value of the *J-V* curve. By holding a bias near the maximum power output point (1.00 V), we obtained a stabilized photocurrent of 23.3 mA cm^−2^, corresponding to a stabilized efficiency of 21.70% (Fig. 3e). Similarly, the photovoltaic performance of PTAA-based devices with gelated HTL strategy is also improved (Fig. S17). This demonstrates the universality of the strategy.

To assess their stability, we carried out a series of investigations on unencapsulated planar devices. As mentioned above, LiTFSI in spiro-OMeTAD are necessary to improve photovoltaic performance, but the hygroscopicity of LiTFSI accelerates the degradation of the perovskite layer. When keeping the devices in an ambient atmosphere (RH of ≈30–60%, 25 °C), the Reference devices exhibited a dramatically decreased PCE, retaining only 60% of their initial PCE values after 1000 h of exposure (Figs. 3f and S18), because the porous morphology ascribed to the tBP volatilization and LiTFSI aggregation created pathways for moisture evasion [43]. Encouragingly, the Target devices retained 92% of their initial PCE in the same condition, even after 2500 h, which could be ascribed to the enhanced stability of the gelation of HTL. Moreover, we found that the unencapsulated Target devices also exhibited promising long-term stability under high RH of 85–90% (Figs. 3g and S19), where 85% of their initial PCEs were retained after 1000 h, whereas the Reference devices only maintained 75% of their initial PCEs after 530 h of exposure.

We further examined the operational stability of the devices under continuous 1-Sun equivalent white LED illumination in nitrogen at room temperature by tracking their MPPs. As shown in Fig. 3h, the Target device maintained over 85% of their initial PCE after 1000 h, while Reference devices retained only about 40% of their initial PCE after continuous illumination after 600 h. This suggests that the less agglomeration of LiTFSI in the HTL in Target devices contributes to a higher degree of operational stability because of the strong coordination interaction of the Li^+^ with the TA of the gelated HTL. The above experiments confirm that the gelated HTL strategy significantly improved the long-term stability of PSCs.

### Improved Photovoltaic Performance

**Fig. 4:**
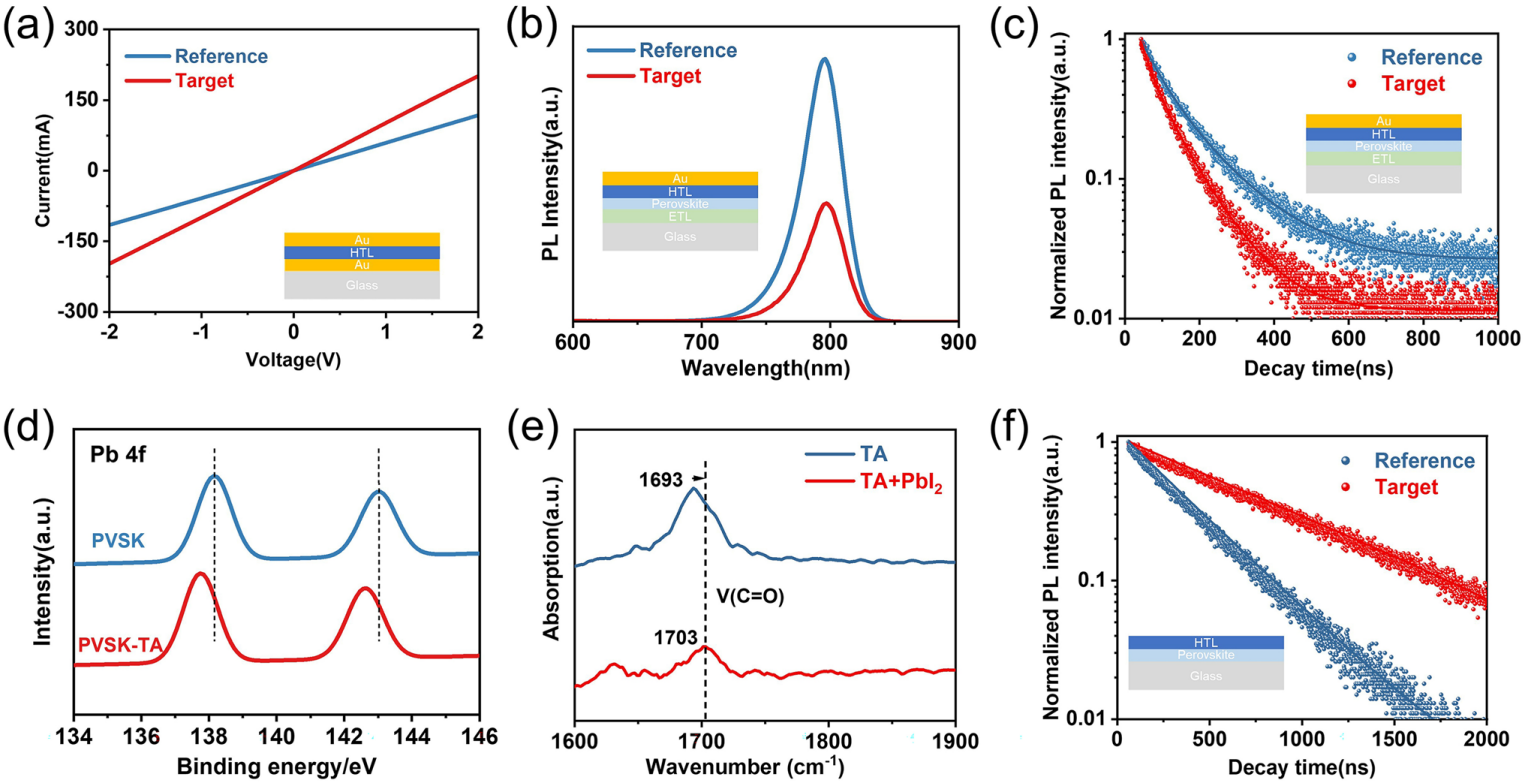
**a** *I–V* curves of ITO/spiro-OMeTAD/Au and ITO/spiro-OMeTAD doped with TA/Au resistance devices. **b** PL curves of Reference and Target perovskite films with HTL. **c** TRPL decay curves of Reference and Target perovskite films with HTL. Note that the TRPL and PL for samples with HTL were measured at a short circuit. **d** XPS spectra of Pb 4*f* of the perovskite and perovskite/TA films. **e** FTIR spectra of TA and TA with PbI_2_ powders. **f** TRPL decay curves of Reference and Target perovskite films with HTL. Note that the TRPL for samples with HTL was measured at an open circuit

To better reveal the origin for the higher efficiency and stability in the gelated HTL devices, the electrical conductivity of spiro-OMeTAD and the gelated HTL films is investigated. Figure 4a shows the current–voltage (*I–V*) relation of ITO/spiro-OMeTAD/Au and ITO/spiro-OMeTAD(TA)/Au resistance devices, from which the resistance *R* can be obtained to calculate the conductivity *σ*. The calculated *σ* is 1.39 × 10^−6^  S cm^−1^ for fresh gelated HTL film, higher than that of fresh spiro-OMeTAD film (6.31 × 10^−7^  S cm^−1^). Clearly, the presence of TA enhances greatly the electrical conductivity of spiro-OMeTAD film. As reported in previous studies [44], S atom in TA has a strong attraction to the electron cloud on C atom due to its strong electronegativity, which facilitates the oxidation spiro-OMeTAD material (Fig. S20). Therefore, TA-doped HTL shows a higher conductivity therein, in comparison with pristine spiro-OMeTAD.

To get insight into the effects of the gelated HTL film on the device performance, the carrier behavior at the perovskite/HTL interfaces is studied by the steady-state photoluminescence (PL) and time-resolved photoluminescence (TRPL) spectroscopy at short circuit condition (Fig. 4b, c). The Target film shows a significantly lower PL intensity (Fig. 4b), indicating that gelated HTL therein effectively promotes the transfer and extraction of photogenerated holes at the perovskite/spiro-OMeTAD interface, which is further confirmed by the TRPL decay (Fig. 3c). The *V*_OC_ dependence on light intensity measurement was conducted further. As shown in Fig. S21, and the ideality factors were calculated as 1.49 and 1.27 for the Reference and Target, respectively. Then, the space charge limited current (SCLC) measurement (Fig. S22) exhibited enhanced carrier mobility of the gelated HTL. This reduction of n_trap_ is attributed to the less defects on the surface of perovskite films induced from the gelated HTL passivation.

To further understand whether the gelated HTL influences the notorious trap states mainly localized at the perovskite surface, X-ray photoelectron spectroscopy of the perovskite film coated with and without TA solution (1 mg mL^−1^) was conducted (Fig. 4d). The remarkable shift in the Pb 4*f* peaks of the perovskite film to lower binding energy after coating with TA indicates that a strong interaction occurred between TA and the perovskite. This behavior was further confirmed by FTIR measurements, where the stretching vibration mode of C = O (Fig. 4e) of the TA showed a remarkable shift to higher fields after mixing with PbI_2_. This result demonstrates that the interaction between TA and the under-coordinated Pb^2+^ defects. Moreover, the PL and TRPL of the reference and target films on the glass were also measured at open circuit condition to verify the defect passivation of the perovskites covered with the gelated HTL. Figure 4f shows that the average PL lifetime of target perovskite film was longer than that of reference perovskite film. The longer PL lifetime generally indicated fewer defects in the perovskites and thus suppressed nonradiative recombination of charge carriers, leading to a strong PL intensity in the spectrum (Fig. S23). Therefore, the gelated HTL strategy not only improved the transport of carriers, but also reduced the defects of the surface of perovskite films.

## Conclusion

In summary, we demonstrate an effective approach by modifying spiro-OMeTAD layer with TA to gel formation in HTL for PSCs. Compared with the conventional spiro-OMeTAD, the gelated HTL displays more uniform and compact film morphology. Furthermore, the gelated HTL shows a more persistent of high humidity due to the inhibition of the LiTFSI aggregation. In addition, TA can effectively passivate the defect states of perovskite films and accelerating the charge transfer from perovskite layer to HTL. As the result, more than 22% of PCE is achieved by employment of this gelated HTL. The PSC with gelated HTL also displays a better stability, which retain 85% of their initial PCE after 1000 h of continuous light aging at 25 °C and 92% of their initial PCE 2500 h under 25 °C in an ambient environment. Our results provide a promising and simple route to improve spiro-OMeTAD-based HTL for the efficient PSCs with a high long-term stability.

### Supplementary Information

Below is the link to the electronic supplementary material.

Supplementary file1 (PDF 1419 KB)
