# Supplementary material for: Gelation of Hole Transport Layer to Improve the Stability of Perovskite Solar Cells
Source: Nanomicro Lett. 2023 Jul 10;15:175. doi: 10.1007/s40820-023-01145-y (PMC10333165; doi:10.1007/s40820-023-01145-y)
Supplement: Supplementary file 1 — Supplementary file1 (PDF 1419 KB) [file 40820_2023_1145_MOESM1_ESM.pdf]

Supporting Information for

Gelation of Hole Transport Layer to Improve the Stability of Perovskite Solar Cells

Ying Zhang[1], Chenxiao Zhou[1], Lizhi Lin[1], Fengtao Pei[1], Mengqi Xiao[1], Xiaoyan Yang[1], Guizhou Yuan[1], Cheng Zhu[1], Yu Chen[1] and Qi Chen[1, *]

[1]Beijing Key Laboratory of Construction Tailorable Advanced Functional Materials and Green Applications, MIIT Key Laboratory for Low-dimensional Quantum Structure and Devices, Experimental Center of Advanced Materials, School of Materials Science and Engineering, Beijing Institute of Technology, Beijing 100081, P. R. China.

*Corresponding author. E-mail: qic@bit.edu.cn (Qi Chen)

Supplementary Figures

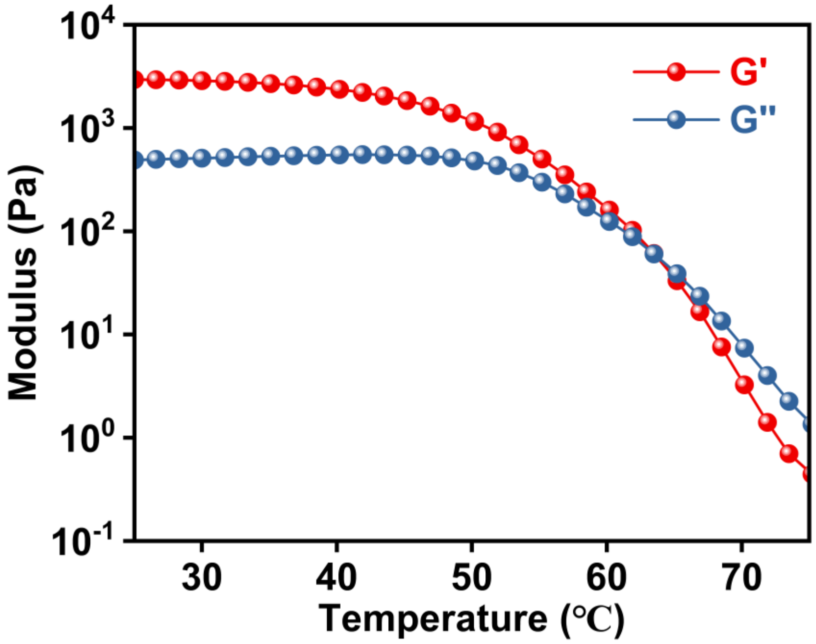

Fig. S1 Storage modulus (G′) and loss modulus (G″) for poly(TA) gels on temperatures sweep

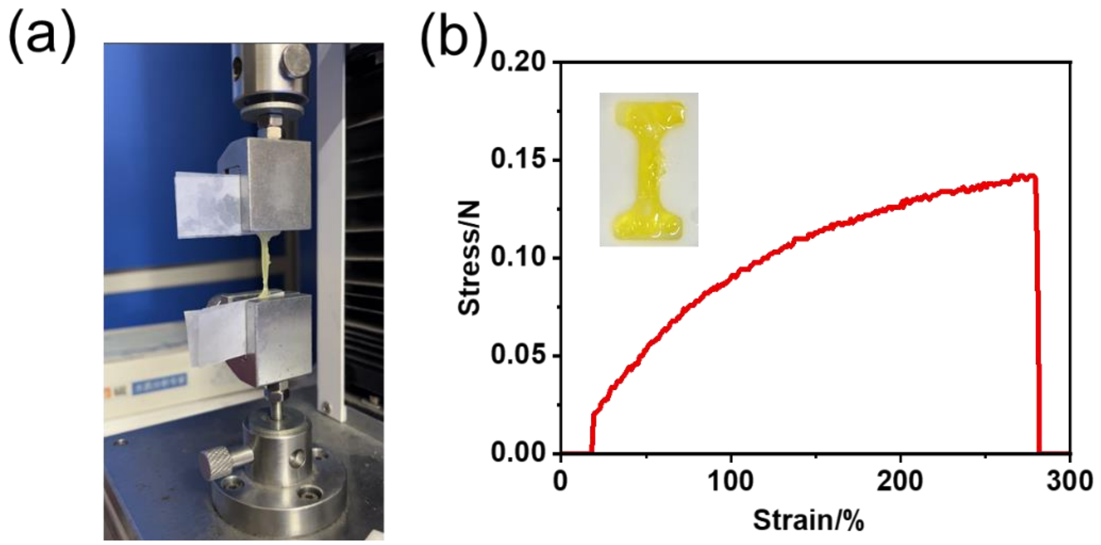

Fig. S2 a The picture of the tensile test. **b** Stress-strain curves of the poly(TA) gel. The picture of sample for tensile test is shown in the inset

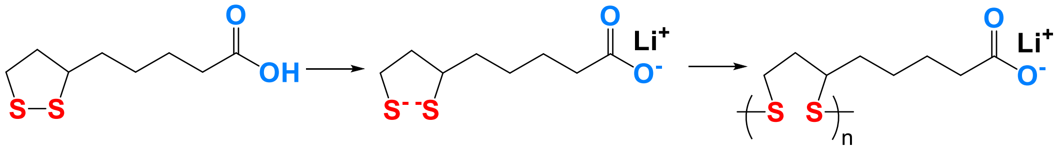

Fig. S3 Synthetic route of TA to poly(TA) during gelation of HTL

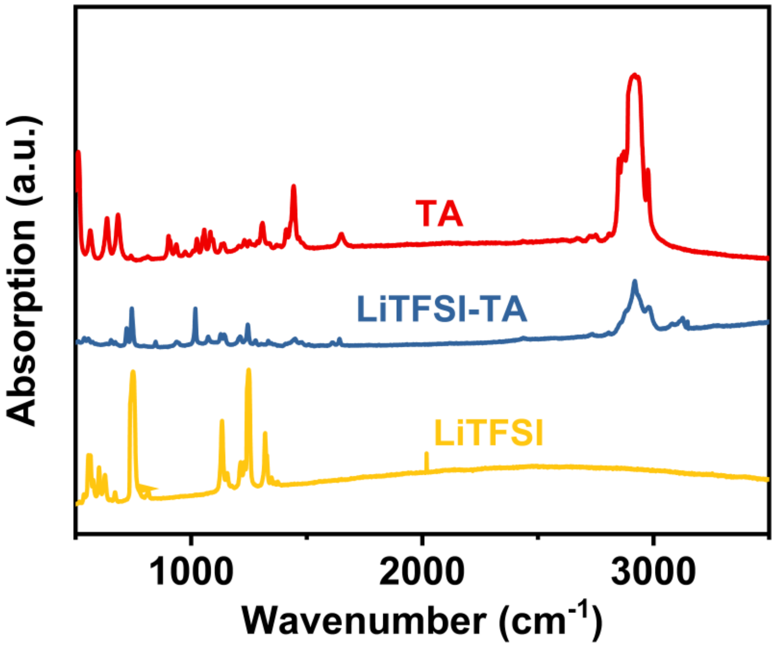

Fig. S4 Full FTIR spectra of a TA (red), mixture of LiTFSI and TA (blue) and LiTFSI (yellow)

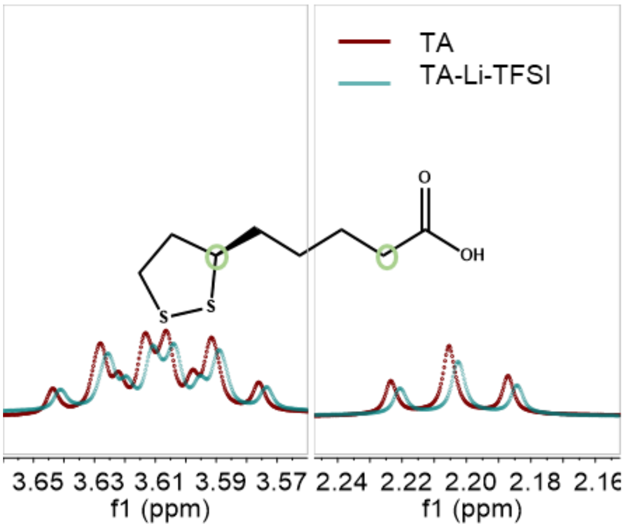

Fig. S5 Partial ^{1}H-NMR spectra of TA-LiTFSI mixture

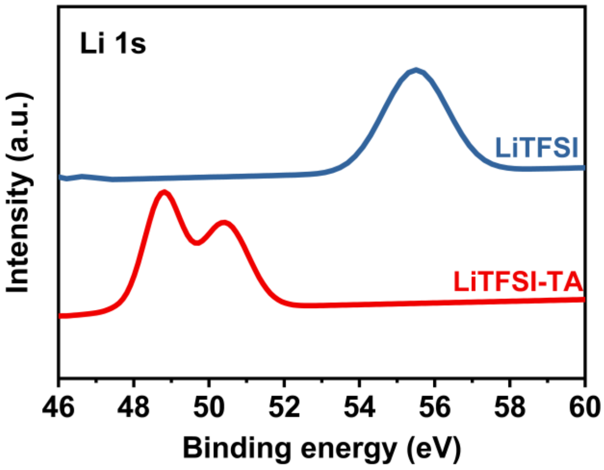

Fig. S6 XPS spectra of Li 1s for LiTFSI and mixture of LiTFSI and TA

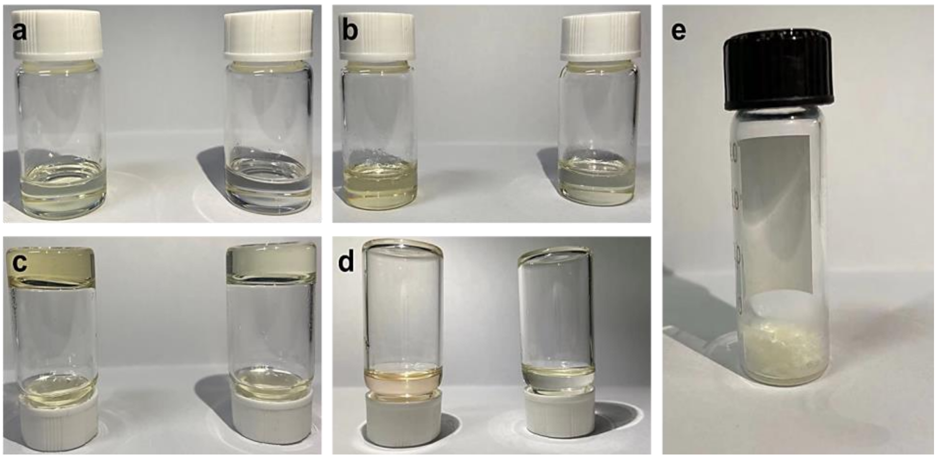

Fig. S7 The pictures of **a** HTL precursor solution with Spiro-OMeTAD (left) or without Spiro-OMeTAD (right). **b** Gel formation after adding TA. **c** Complete gelation. **d** Sol after adding ethanol. **e** Dry gel state after solvent evaporation.

Supplementary discussion: The HTL precursor solution would immediately be gelated while adding TA into solution. Figure S7 shows the gelation and solification process of the gelated HTL precursor and the dry gel state after removal of volatile solvent. Figure S7a is the precursor solution of the Spiro-OMeTAD based HTL. The bottle on the left contains Spiro-OMeTAD and the bottle on the right does not. Figure S7b shows the phenomenon after adding TA to the solution. Gel was formed almost instantaneously. When we let it sit for a few minutes, the whole solution is gelated. As shown in Fig. S7c, at this time, after we turn the bottle upside down, the gel will not fall. However, the formed gel state cannot be directly used for spin-coating process. It was found that volatile formic acid or ethanol could dissolve the gel. Therefore, when a small amount of volatile acid or alcohol is added, the gel can be completely dissolved, as shown in Fig. S7d. Figure S7e shows the state of sol after complete volatilization of volatile solvent at low temperature. After preparing the sol, the subsequent spin coating operation can be continued. After depositing the sol-state precursor, some volatile components such as swelling agent chlorobenzene, tBP, etc. will volatilize, and form a xerogel containing Spiro-OMeTAD molecules in finally. In this process, we did not filter the solution using a PTFE filter in order not to disrupt the gelation process.

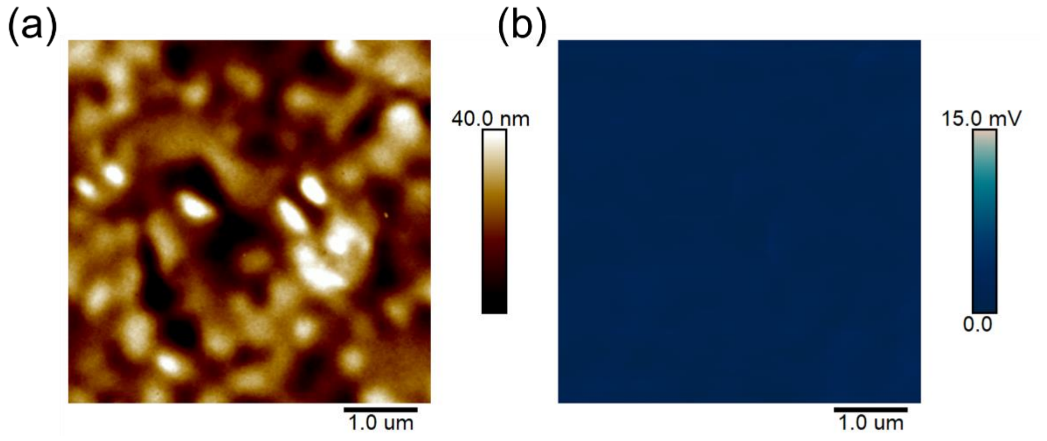

Fig. S8 AFM images of Reference film (**a**) and corresponding Nano-FTIR images (**b**). Nano-FTIR at an IR frequency of 1693 cm^{-1}(which is resonant with the C=O stretching absorption of TA)

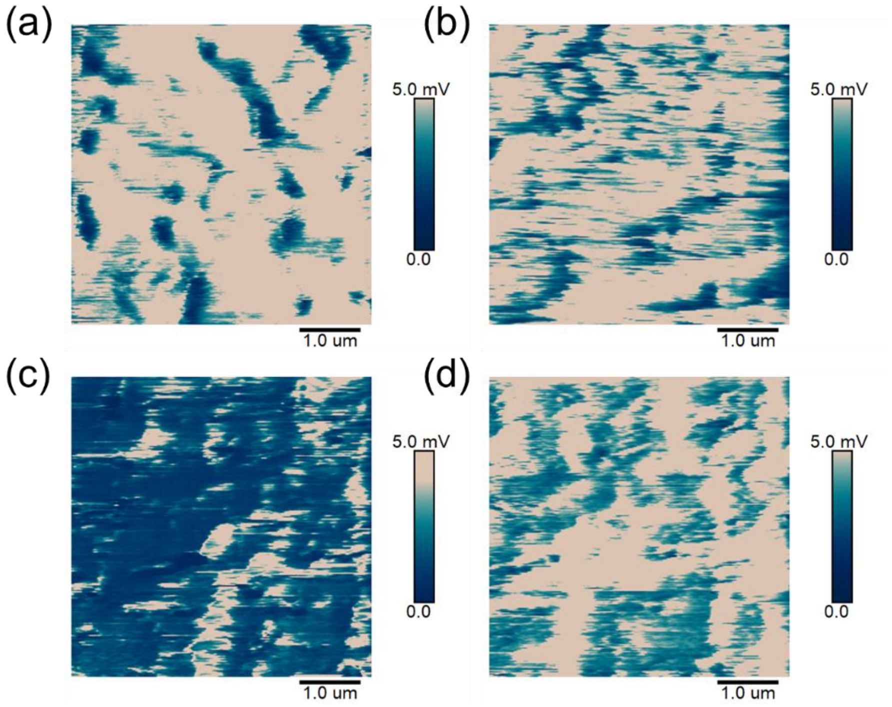

Fig. S9 Corresponding Nano-FTIR images of Reference film (**a,c**) and Target film (**b,d**) before aged and after aged under a relative humidity (RH) of 85% at 25 °C. Nano-FTIR at an IR frequency of 1264 cm^{-1}(which is resonant with the CF_3 stretching absorption of LiTFSI)

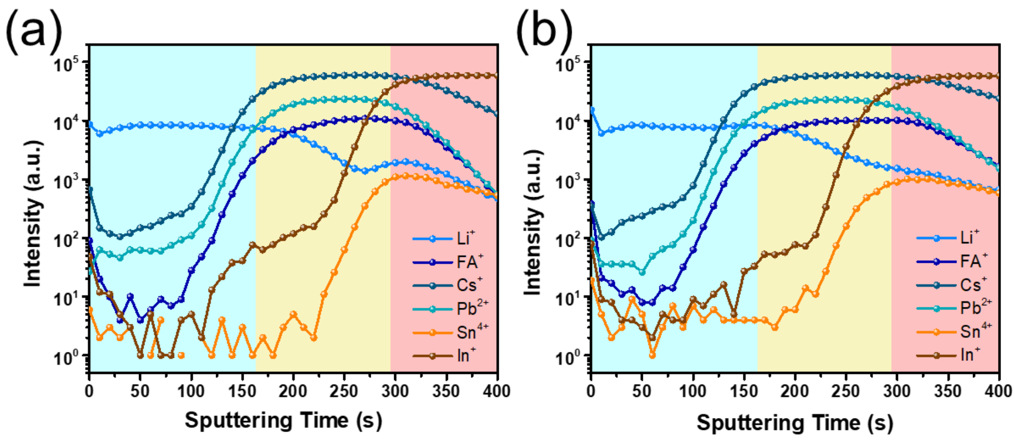

Fig. S10 ToF-SIMS depth profiles of the Reference (**a**) and Target device (**b**) after aged under an RH of 85% at 25 °C

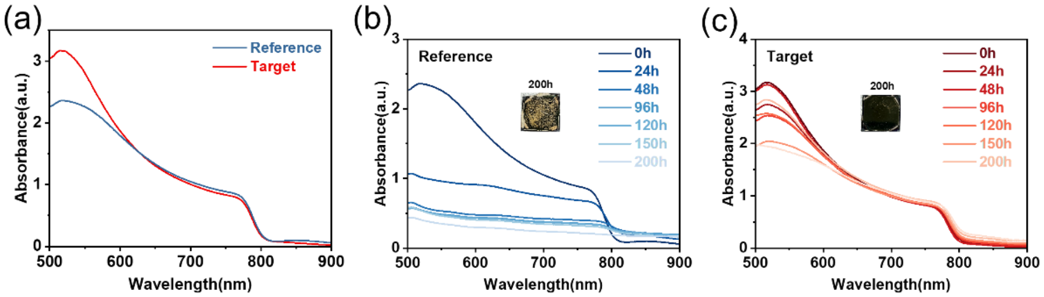

Fig. S11 UV-vis absorption of fresh film (**a**), Reference film (**b**) and Target film (**c**) under an RH of 85-90% at 25 °C in dark conditions with respect to the exposure duration

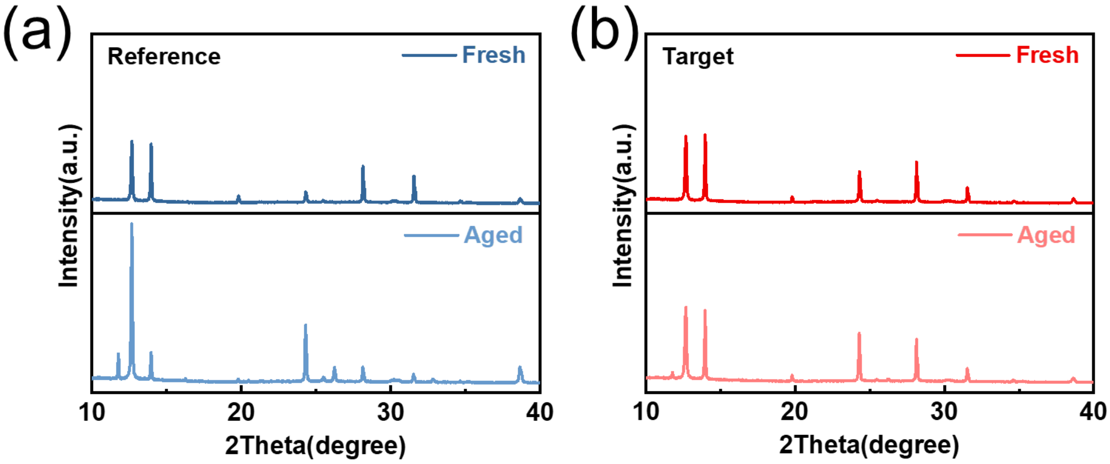

Fig. S12 XRD spectra for Reference and Target films before and after aged under an RH of 85-90 % at 25 °C for 200 h

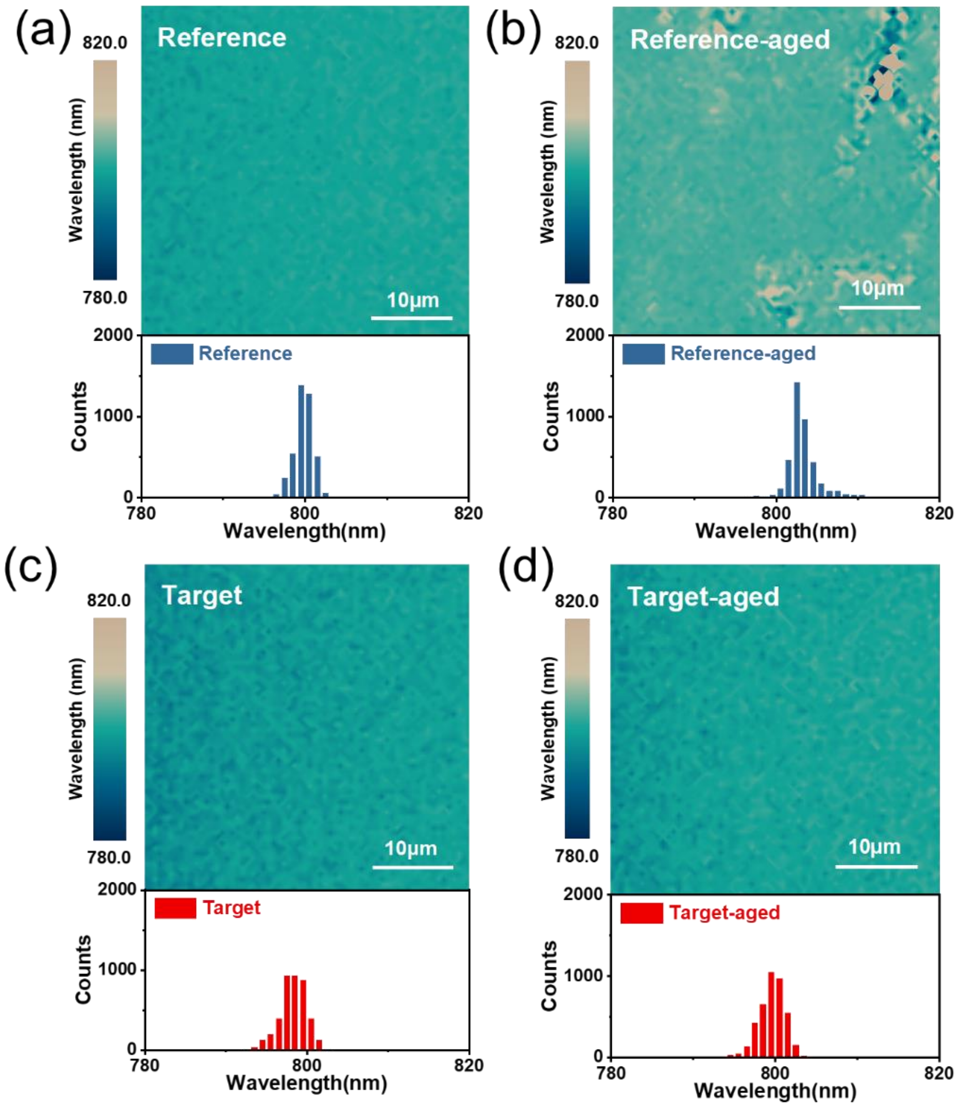

Fig. S13 a PL peak position mapping and statistical diagram of Reference and **b** Target films before and after aging under high RH of 85-90 % at 25 °C for 500 h

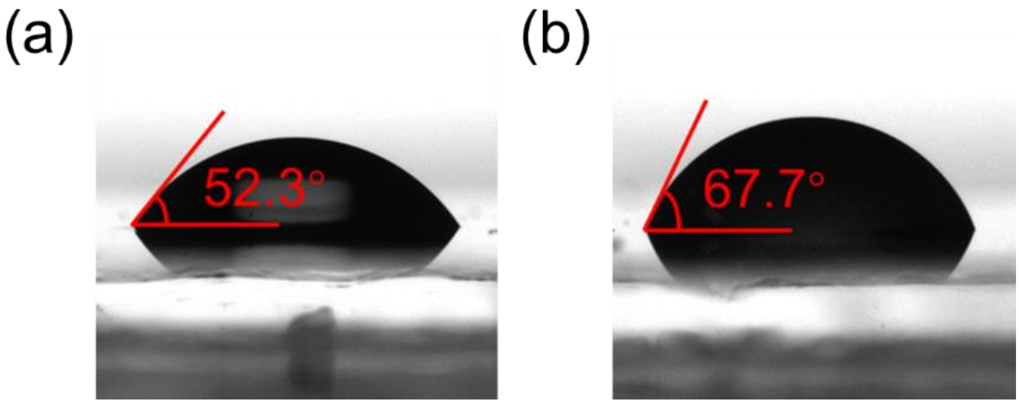

Fig. S14 Contact angle measurement of a water droplet on the surfaces of the Reference HTL (**a**) and the gelated HTL (**b**)

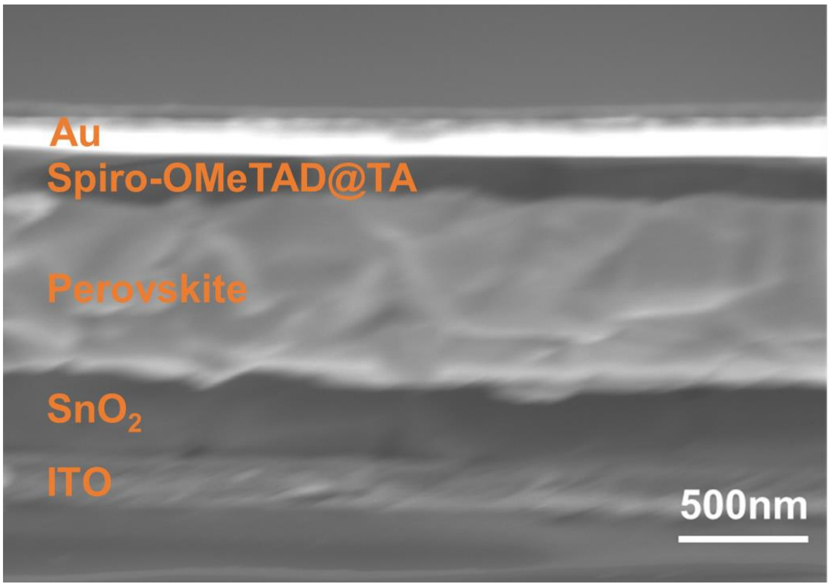

Fig. S15 Cross-sectional SEM images of the Target device

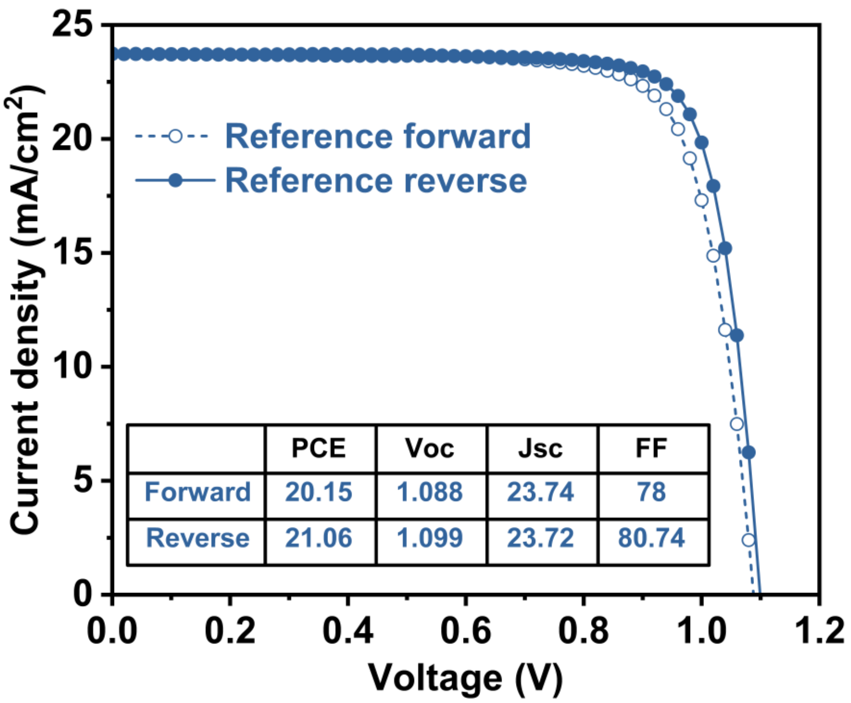

Fig. S16 *J-V* curves with reverse (1.2 V to −0.2 V) and forward (−0.2 V to 1.2 V) scan of Reference devices with aperture areas of 0.0805 cm^2

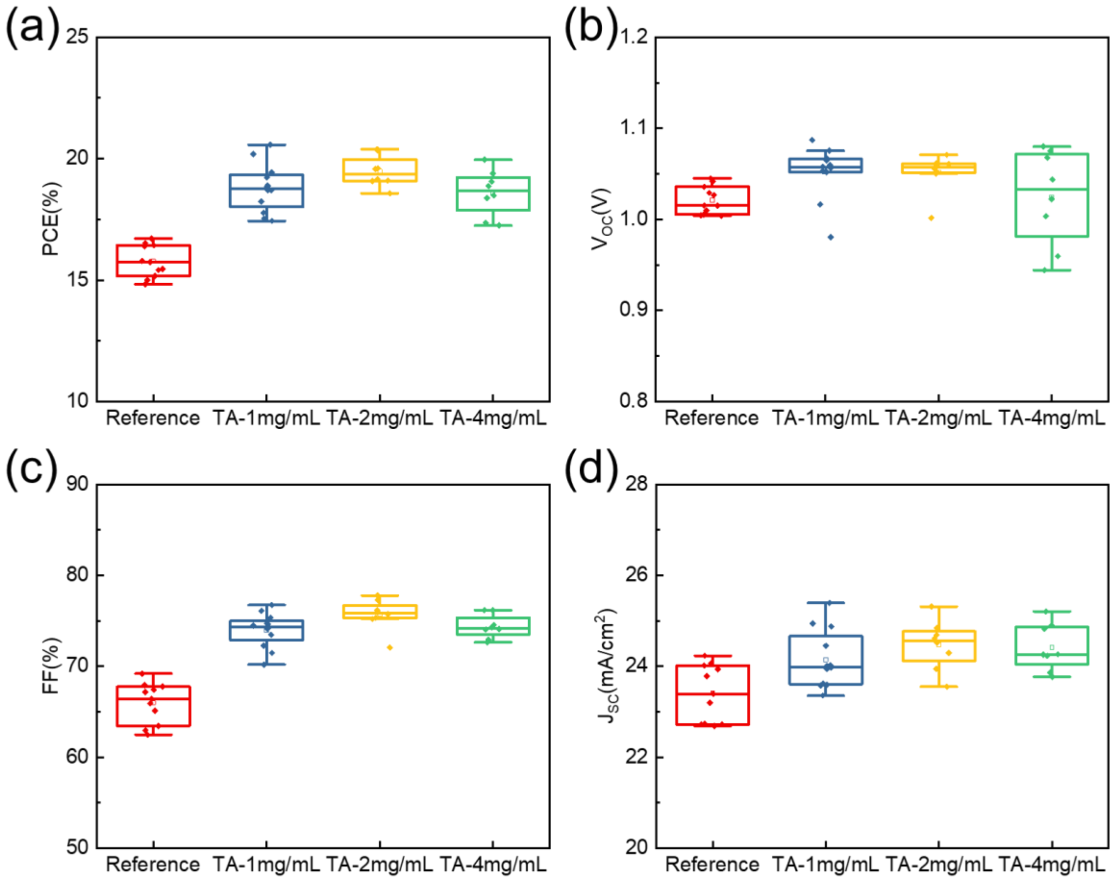

Fig. S17 Photovoltaic performance of PTAA-based PSCs with different concentrations of TA

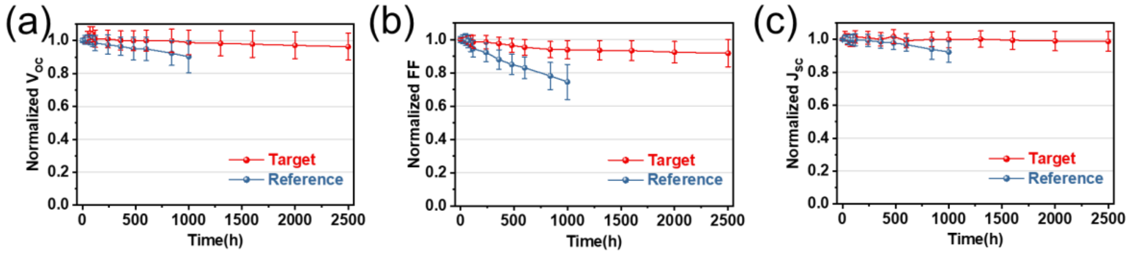

Fig. S18 Photovoltaic performance parameters of devices as a function in air environment with a humidity of about 30–60% at a temperature of about 20 ℃

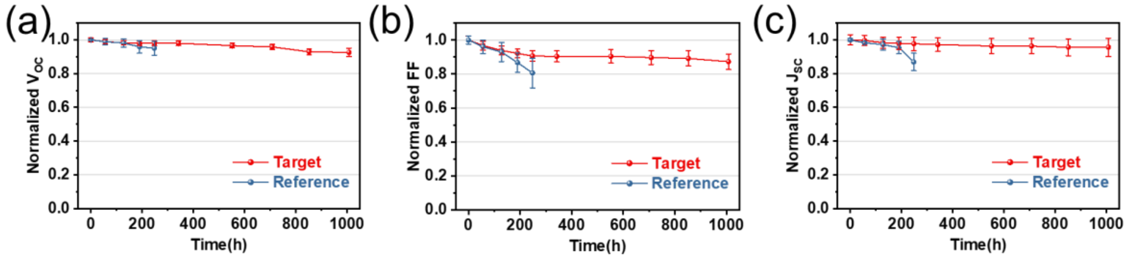

Fig. S19 Photovoltaic performance parameters of devices as a function in 85% RH at a temperature of about 20 ℃

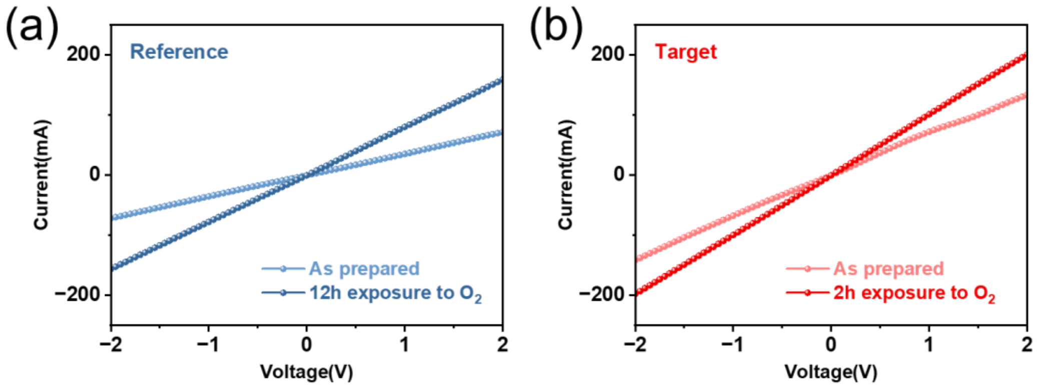

Fig. S20 *I–V* curves of ITO/spiro-OMeTAD/Au (**a**) and ITO/spiro-OMeTAD doped with TA/Au (**b**) resistance devices as prepared and after exposure to O_2

Supplementary discussion: The oxidation of spiro-OMeTAD is promoted by LiTFSI [S1, S2]. In the initial oxidation of spiro-OMeTAD by dioxygen, oxygen radicals could form. Subsequently, these oxygen radicals are removed by LiTFSI. The remaining Li cation forms a Li_xO_y species, and the $TFSI^-$ anion traps/stabilizes the oxidized spiro-$OMeTAD^+$ [S3]. An important parameter in the oxidation process is the oxidation time. In the literature, it is stated that the oxidation of spiro-OMeTAD generally requires 8–12 h, and in some cases, overnight [S4]. As the oxidation time increases, the conductivity of spiro-OMeTAD films increases accordingly [S5, S6]. The reason for the increase in conductivity of TA-doped HTL is possible that the molecular interaction between TA and spiro-OMeTAD plays an important role. As reported in previous studies [7], S atom in TA has a strong attraction to the electron cloud on C atom due to its strong electronegativity, which plays the role of oxidizing spiro-OMeTAD material. We fabricated hole-only devices (ITO/HTL/Au) with different oxidation time and detected the current–voltage *(I-V)* curves under the dark state to investigate the effect of gelation of HTL films (**Fig. S20**). As-prepared spiro-OMeTAD films showed conductivity values of 6.31×10^{-7} S·cm^{-1}. After 12 h exposure to O_2, samples showed an apparently increase in conductivity (1.39×10^{-6} S·cm^{-1}) (**Fig. S20a**). For the as-prepared gelated HTL film, higher conductivity than the pristine spiro-OMeTAD film was shown (1.25×10^{-6} S·cm^{-1}). Moreover, after only 2 h exposure to O_2, the conductivity of the gelated HTL film (1.76×10^{-6} S·cm^{-1}) can be comparable to that of the spiro-OMeTAD film after 12h oxidation (**Fig. S20b**). This indicated that TA-doped HTL promoted the oxidation process of spiro-OMeTAD.Therefore, TA-doped HTL shows a higher conductivity therein, in comparison with pristine spiro-OMeTAD.

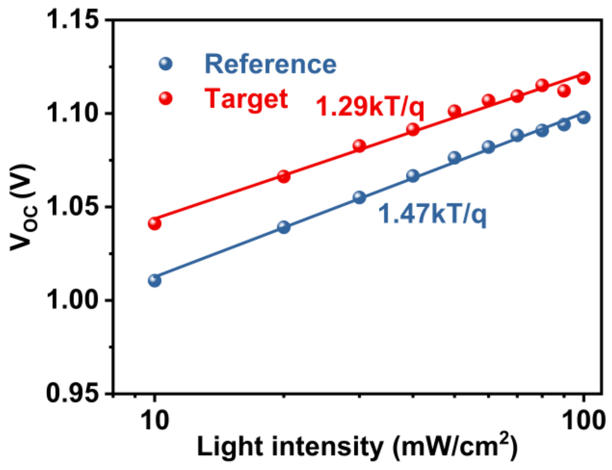

Fig. S21 Light intensity dependence of the V_{OC} of Reference and Target PSCs, where k is the Boltzmann constant and T is the absolute temperature

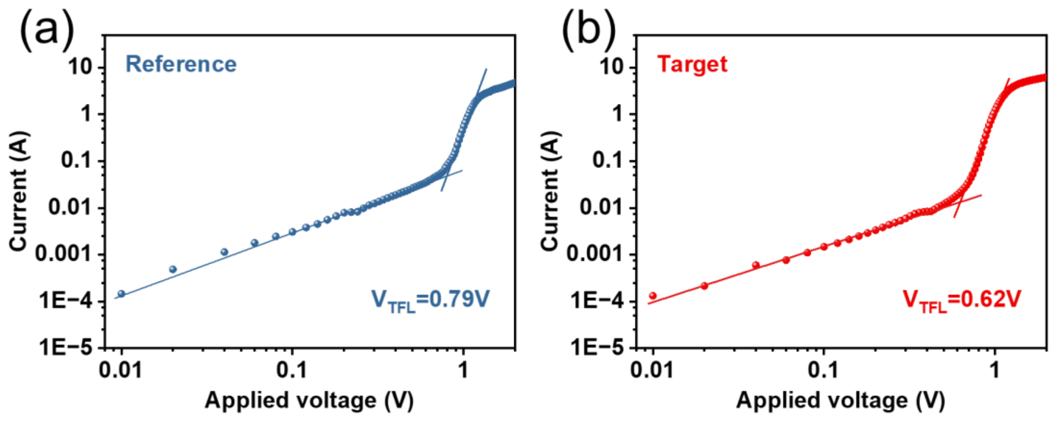

Fig. S22 SCLC characteristics of the ITO/PTAA/Perovskite/HTL/Au device for Reference and Target

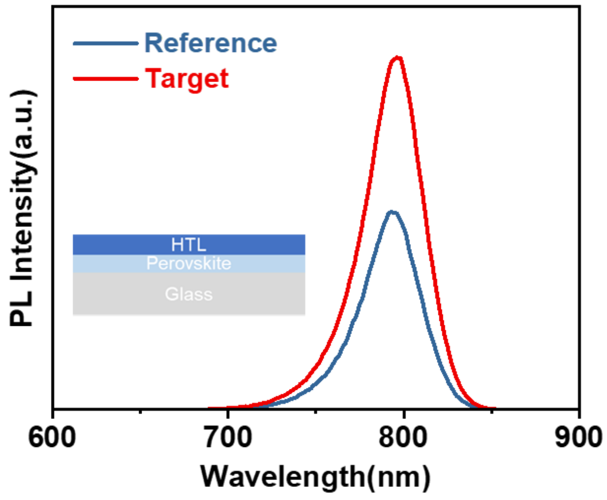

Fig. S23 PL curves of Reference and Target perovskite films with HTL. Note that the TRPL for samples with HTL were measured at an open circuit

Supplementary References

[S1] U. B. Cappel, T. Daeneke, U. Bach, Oxygen-induced doping of spiro-OMeTAD in solid-state dye-sensitized solar cells and its impact on device performance. Nano Lett. **12**(9), 4925-4931 (2012). https://doi.org/10.1021/nl302509q

[S2] Z. Hawash, L. K. Ono, Y. Qi, Moisture and oxygen enhance conductivity of LiTFSI-doped spiro-OMeTAD hole transport layer in perovskite solar cells. Adv. Mater. Interfaces **3**(13), 1600117 (2016). https://doi.org/10.1002/admi.201600117

[S3] A. Abate, T. Leijtens, S. Pathak, J. Teuscher, R. Avolio et al., Lithium salts as "redox active" p-type dopants for organic semiconductors and their impact in solid-state dye-sensitized solar cells. Phys. Chem. Chem. Phys. **15**(7), 2572-2579 (2013). https://doi.org/10.1039/C2CP44397J

[S4] M. Liu, M. B. Johnston, H. J. Snaith, Efficient planar heterojunction perovskite solar cells by vapour deposition. Nature **501**(7467), 395-398 (2013). https://doi.org/10.1038/nature12509

[S5] C. Ding, R. Huang, C. Ahläng, J. Lin, L. Zhang et al., Synergetic effects of electrochemical oxidation of spiro-OMeTAD and Li^+ ion migration for improving the performance of n–i–p type perovskite solar cells. J. Mater. Chem. A **9**(12), 7575-7585 (2021). https://doi.org/10.1039/D0TA12458C

[S6] R. L. Forward, K. Y. Chen, D. M. Weekes, D. J. Dvorak, Y. Cao et al., Protocol for quantifying the doping of organic hole-transport materials. ACS Energy Lett. **4**(10), 2547-2551 (2019). https://doi.org/10.1021/acsenergylett.9b01766

[S7] Q. Du, Z. Shen, C. Chen, F. Li, M. Jin et al., Spiro-OMeTAD:Sb_2S_3 hole transport layer with triple functions of overcoming lithium salt aggregation, long-term high conductivity, and defect passivation for perovskite solar cells. Sol. RRL **5**(11), 2100622 (2021). https://doi.org/10.1002/solr.202100622
